# Draft Genome Sequence of *Piscirickettsia litoralis*, Isolated from Seawater

**DOI:** 10.1128/genomeA.01252-16

**Published:** 2016-11-03

**Authors:** Xuehua Wan, Alex J. Lee, Shaobin Hou, Blake Ushijima, Yen P. Nguyen, Jessica A. Thawley, Stuart P. Donachie

**Affiliations:** a Advanced Studies in Genomics, Proteomics and Bioinformatics, University of Hawai‘i at Mānoa, Honolulu, Hawai‘i, USA; b Department of Microbiology, University of Hawai‘i at Mānoa, Honolulu, Hawai‘i, USA

## Abstract

One species of *Piscirickettsia*, a pathogen of salmonid fish, has been described. The genome sequence of a putative second and free-living species may provide insights into the evolution of pathogenicity in the genus.

## GENOME ANNOUNCEMENT

Strain Y2 was cultivated from seawater at Waimanalo Beach Park, O‘ahu, Hawai‘i. The nearest neighbor based on 16S rRNA gene nucleotide identity is *Piscirickettsia salmonis* LF-89^T^ (97.3%), an economically important obligate intracellular pathogen of salmonids (1, 2). We sequenced the genome of Y2 to investigate the evolutionary relationship between free-living Y2 and the pathogenic *P. salmonis* LF89^T^ and to provide insights into their lifestyles.

Genomic DNA was isolated from Y2 with the Wizard genomic DNA purification kit (Promega, USA), additional cetyltrimethylammonium bromide (CTAB) and phenol extraction steps, and a 30% ethanol wash to remove excess exopolysaccharides. Roche 454 GS FLX+ pyrosequencing generated 66.6 Mb of shotgun sequences and 29.1 Mb of 8-kb paired-end sequences. Newbler 2.8 built 16 scaffolds spanning 3,897,429 bp (scaffold N_50_, 3,047,478 bp), with 263 contigs oriented in the scaffolds. The G+C content is 39.9%.

The Y2 genome was annotated in the NCBI’s Prokaryotic Genome Annotation Pipeline (PGAP), Rapid Annotation using Subsystem Technology (RAST), and Prokka 1.11 (3–6). PGAP identified 3,198 protein-coding genes, 48 tRNA-coding regions, and 576 pseudogenes. RAST identified 4,095 protein-coding genes and 381 subsystems. The Y2 genome is ~11% larger and encodes ~16% more genes than that of *P. salmonis* LF-89^T^, yet it contains one-sixth the number of virulence, disease, and defense genes, as annotated in RAST (7). A total of 4,266 proteins annotated by Prokka were run in BLASTP against Vir/Dot/Icm proteins of the type IV secretion system (T4SS) in the T346Hunter database (8); 4 proteins related to vir-T4SS assembly, including VirB4, VirB9, VirB11, and PtlG, were initially predicted (*E* < 1e^10^). The Y2 proteome lacks homologs of most Vir proteins in the vir-T4SS core components, but 69 proteins were initially predicted to be homologs in Dot/Icm T4SS (*E* < 1e^-10^). Characterization of Y2 and further analysis of the genome will determine if the strain represents the second *Piscirickettsia* species and shed light on the strain’s lifestyle and evolution of *Piscirickettsia* species.

### Accession number(s).

This whole-genome shotgun project has been deposited at DDBJ/EMBL/GenBank under accession number MDTU00000000. The version described here is the first version, MDTU01000000. The 16S ribosomal gene sequence has been deposited at GenBank under accession number KX757850.
